# Pathophysiological Responses and Roles of Astrocytes in Traumatic Brain Injury

**DOI:** 10.3390/ijms22126418

**Published:** 2021-06-15

**Authors:** Shotaro Michinaga, Yutaka Koyama

**Affiliations:** 1 Department of Pharmacodynamics, Meiji Pharmaceutical University, 2-522-1 Noshio, Kiyose, Tokyo 204-8588, Japan; michisho@my-pharm.ac.jp; 2 Laboratory of Pharmacology, Kobe Pharmaceutical University, 4-19-1 Motoyama-Kita Higashinada, Kobe 668-8558, Japan

**Keywords:** astrogliosis, traumatic brain injury, neuroinflammation, cytotoxic edema, blood–brain barrier

## Abstract

Traumatic brain injury (TBI) is immediate damage caused by a blow to the head resulting from traffic accidents, falls, and sporting activity, which causes death or serious disabilities in survivors. TBI induces multiple secondary injuries, including neuroinflammation, disruption of the blood–brain barrier (BBB), and brain edema. Despite these emergent conditions, current therapies for TBI are limited or insufficient in some cases. Although several candidate drugs exerted beneficial effects in TBI animal models, most of them failed to show significant effects in clinical trials. Multiple studies have suggested that astrocytes play a key role in the pathogenesis of TBI. Increased reactive astrocytes and astrocyte-derived factors are commonly observed in both TBI patients and experimental animal models. Astrocytes have beneficial and detrimental effects on TBI, including promotion and restriction of neurogenesis and synaptogenesis, acceleration and suppression of neuroinflammation, and disruption and repair of the BBB via multiple bioactive factors. Additionally, astrocytic aquaporin-4 is involved in the formation of cytotoxic edema. Thus, astrocytes are attractive targets for novel therapeutic drugs for TBI, although astrocyte-targeting drugs have not yet been developed. This article reviews recent observations of the roles of astrocytes and expected astrocyte-targeting drugs in TBI.

## 1. Introduction

Traumatic brain injury (TBI) is severe damage to the brain and is referred to as a sudden insult caused by traffic accidents, falls, and sporting activity. TBI is a primary cause of unexpected death or induces serious disabilities, including motor and cognitive dysfunction, in survivors. Over 10 million young and old people experience TBI worldwide per year. TBI elicits dysfunction in the brain environment, including neuronal circuits and cerebral vascular functions. As treatments for TBI, decompressive craniotomy, hyperosmolar treatment, barbiturate, sedation, and hypothermia therapy [1] are performed to reduce intracranial pressure (ICP) in acute TBI patients. However, these therapies are insufficient in some cases and have significant adverse effects. Some rehabilitations are also performed to improve motor and cognitive functions in patients with chronic TBI. To date, many candidate therapeutic drugs have been discovered in preclinical studies using experimental TBI animal models, and some of them have been examined in clinical trials. Although preclinical studies have suggested that some candidates show promising beneficial actions, clinical trials have failed to show significance in patients with TBI [2,3,4]. Most of the investigated candidate drugs were targeted to an individual injury factor, neuronal cells, or cerebral vasculature. However, emerging evidence shows that the pathogenesis of TBI is induced by multiple injury factors, and glial cells also play significant roles in the pathogenesis of TBI.

Astrocytes are key players in the pathogenesis of neurodegenerative disorders and brain injury. In the damaged brain, astrocytes convert to the reactive form from the resting form, and reactive astrocytes exert both protective and detrimental functions. Multiple studies have suggested that some preclinical drugs exert beneficial effects on neuronal damage by promoting or attenuating astrocyte functions in experimental animal models. Additionally, Qian et al. showed conversion of midbrain astrocytes to dopaminergic neurons, which provide axons to reconstruct the nigrostriatal circuit in Parkinson’s disease model mice, suggesting a novel approach to treating neurodegeneration by replacing lost neurons [5]. Thus, astrocytes are attractive targets for therapeutic drugs in brain disorders and injuries. 

In patients with TBI, increased reactive astrocytes are observed in damaged areas [6]. A recent study found that levels of astrocyte-derived neurotoxic exosome complement proteins that elicit damage to synapses and injuring neurons were also increased in TBI patients [7]. Emerging evidence suggests that astrocytes elicit beneficial and detrimental roles in the pathogenesis of TBI, including neuroinflammation, brain edema, disruption of the blood–brain barrier (BBB), neurogenesis, and synaptogenesis [8,9,10]. In this review, recent observations of the roles of astrocytes in TBI and expected candidate therapeutic drugs targeted to astrocytes in TBI are summarized.

## 2. Pathologies of TBI

TBI drives multiple conditions, including axonal damage, neuronal death, gliosis, disruption of the BBB, edema, intracranial hemorrhage, hypoxemia, hypotension, and neuroinflammation [8,9]. On the other hand, neurogenesis and synaptogenesis are also promoted after TBI to recover lost neuronal functions [10]. The severity of TBI ranges from mild to severe. TBI is commonly categorized as focal or diffuse. Focal injury is caused by direct impact and includes scalp injury, skull fracture, and surface contusions, which lead to mechanical focal brain damage and diffuse axonal injury by shearing, tearing, and stretching. On the other hand, diffuse injury results from acceleration–deceleration forces, which include hypoxic–ischemic damage, meningitis, and vascular injury. 

TBI occurs in two different phases: primary and secondary injury. Primary injury results from direct physical impact to the head and immediately causes fatal brain damage, such as contusion and hemorrhage in the injured core area. This results in irreversible neuronal and axonal damage and vascular damage. Following TBI, the brain region surrounding the primary injury is known as a traumatic penumbra as well as stroke and is considered to have the potential to recover [11,12]. This region undergoes secondary injury that includes dysfunction of the BBB, brain edema, and neuroinflammation [13]. Secondary injury is mainly driven by astrocytes, microglia, and infiltrated immune cells from peripheral tissues, and causes continuous neuronal and vascular dysfunction. Following the primary injury, secondary injury occurs from hours to days to months after the initial trauma. Primary injury is inevitable, while delayed development of the secondary injury provides a window of opportunity for therapeutic intervention to prevent progressive damage and improve functional recovery after TBI. The induction of reactive astrocytes is propagated in the brain region where the secondary damage of TBI is spreading. Therefore, many studies have investigated the role of reactive astrocytes in the development of TBI pathologies.

## 3. Conversion to Reactive Astrocytes in TBI

Reactive astrocytes are commonly characterized by structural and functional conversion of astrocytes. The characterized conversions include cell hypertrophy, heightened proliferation, secretion of inflammatory mediators and neurotrophic factors, and increased expression of intermediate filaments such as glial fibrillary acidic protein (GFAP) and vimentin [14,15]. Reactive astrocytes possess a high proliferative ability, referred to as astrogliosis. As transgenic mice lacking GFAP and vimentin have markedly impaired astrocyte reactivity in TBI, these cytoskeletal proteins are essential for the appropriate initiation and maintenance of reactive astrogliosis [16,17]. 

Many studies have suggested that astrocytes convert from the resting to reactive type in both TBI patients and model animals. In TBI patients, increased reactive astrocytes are predominantly observed in damaged areas [6]. Increased GFAP expression was also observed in patients with TBI [18,19,20]. Additionally, S100β, a marker of reactive astrogliosis, was elevated in the serum and cerebrospinal fluid of patients with TBI [21,22]. Similarly, expression of GFAP and vimentin was also increased in several TBI animal models [23,24,25,26,27]. In a mouse controlled cortical impact (CCI), an experimental TBI model, hypertrophic astrocytes in the lesional and peri-lesional areas were observed 3 days after TBI [28]. GFAP-positive astrocytes were found to proliferate at 1, 3, and 7 days post-injury, with numbers of proliferating astrocytes peaking at 3 days post-injury in a mouse CCI model [29]. We also found that GFAP-positive reactive astrocytes were increased in TBI mice following fluid percussion injury (FPI) [30].

Emerging studies suggest that reactive astrocytes play a dual role in TBI. Ablation of proliferating reactive astrocytes after TBI by CCI aggravates inflammation and neuronal death in mice [31]. On the other hand, improved axonal growth and repair following experimental brain and spinal cord injury were demonstrated in transgenic mice deficient in both vimentin and GFAP [16,32]. Thus, reactive astrocytes can have beneficial or detrimental effects following TBI. Based on these findings on the role of reactive astrocytes in the pathology of TBI, controlling the functions of reactive astrocytes is suggested to be a novel therapeutic strategy to improve nerve damage caused by TBI. To establish an effective method to control reactive astrocytes, the mechanisms underlying the conversion to reactive astrocytes and functional alterations have been studied.

## 4. Mechanism of Converting to Reactive Astrocytes

### 4.1. Factors Inducing Reactive Astrocytes 

Converting to reactive astrocytes is triggered by multiple bioactive factors that are increased in the injured area after TBI. In patients with TBI and experimental TBI mice, expression of endothelin-1 (ET-1) was increased [30,33,34], and increased ET-1 promoted conversion to reactive astrocytes via the ET_B_ receptor in TBI mice by FPI [30]. Several inflammatory cytokines and chemokines also trigger astrogliosis. Interleukin-1 (IL-1) promotes conversion to the reactive form of astrocytes [35,36], while an IL-1 receptor antagonist reduced hippocampal astrogliosis in a CCI-induced TBI mouse model [37]. Monocyte chemoattractant protein-1 (MCP-1) promotes astrogliosis via CC chemokine receptor (CCR) [38]. CCR5 knockdown or CCR5 antagonist reduced astrogliosis in the lesioned cortex and reduced the lesion area in TBI mice [39,40]. Additionally, the expression of vascular endothelial growth factor (VEGF) was also increased in FPI-induced TBI model mice [30], and VEGF inhibitor decreased in reactive astrocytes after TBI in mice by suppressing the Toll-like receptor 4/nuclear factor-kappa B (NF-κB) signaling pathway [41]. The reported regulatory factors for reactive astrocytes are summarized in Table 1.

### 4.2. Intracellular Signals Underlying the Conversion to Reactive Astrocytes 

**Figure 1:**
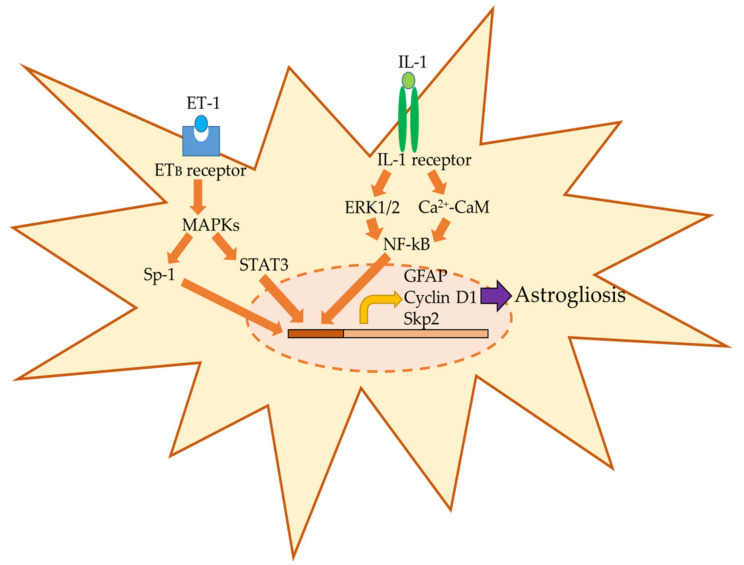
Expected mechanisms on astrogliosis in TBI. TBI promotes the expression of multiple bioactive factors such as endothelin-1 (ET-1) and interleukine-1 (IL-1). ET-1 and IL-1 bind to the ET_B_ receptor and IL-1 receptor in astrocytes, respectively. Stimuli of these receptors activate the mitogen-activated protein kinase (MAPK) and Ca^2+^-calmodulin (CaM) pathways that promote the expression of glial fibrillary acidic protein (GFAP), cyclin D1, and S-phase kinase-associated protein 2 (Skp2) via activation of transcriptional factors including signal transducer and activator of transcription 3 (STAT3), specificity protein-1 (Sp-1), and nuclear factor-κB (NF-κB) in astrocytes, resulting in astrogliosis.

Multiple intracellular signaling pathways control the conversion to reactive astrocytes, and the expected signal mechanisms are summarized in Figure 1. Signal transducer and activator of transcription 3 (STAT3) is closely related to astrogliosis. In FPI-induced TBI rats, confocal microscopy revealed that STAT3 was localized primarily within astrocytic nuclei [42]. We suggest that STAT3-mediated regulation of cell proliferation-related proteins, such as cyclin D1 and S-phase kinase-associated protein 2, underlies ET-induced astrocytic proliferation [43]. Additionally, ET-induced astrocytic proliferation was triggered by the phosphorylation of specificity protein-1, a transcriptional factor involved in the activation of mitogen-activated protein kinase (MAPK) in cultured astrocytes [44]. 

NF-κB is also involved in astrocyte reactivity. Activated NF-κB is primarily localized within astrocytes in brain regions exhibiting reactive gliosis, inflammatory activation, and cellular atrophy following TBI in rats [45,46]. In CCI-induced TBI rats, activation of NF-κB was promoted [47], and transgenic inhibition of astrocytic NF-kB signaling reduced astrogliosis in a mouse model of vascular dementia [48]. Activation of NF-κB also promotes swelling in cultured astrocytes after FPI-induced TBI [49]. Increased GFAP expression by IL-1 is mediated by NF-kB and phosphorylation of extracellular signal-regulated kinase (ERK)1/2, and the NF-kB/Ca^2+^-calmodulin (CaM)/ERK signaling pathway has been suggested as a key regulator of IL-1-induced astrogliosis [50]. Thus, these intracellular signaling pathways control astrogliosis in TBI, although other pathways for astrogliosis may be found in the future.

### 4.3. Role of Chaperone Proteins for Converting Reactive Astrocytes

Chaperones play a key role for the protection of cells from stress, such as an inflammatory response. Recent studies imply that chaperone proteins control astrogliosis. Sigma-1 receptor (Sig-1R) functions as a chaperone and increased GFAP expression was observed in mixed neuronal–glial cultures derived from Sig-1R KO mice [51]. OZP002, a Sig-1R positive modulator, prevented amyloid β25-35-induced reactive astrogliosis in the hippocampus [52]. Heat shock protein 72 (Hsp72) is a chaperone protein and protects from brain injury. Overexpression of Hsp72 reduced the density of GFAP- and vimentin-expressing cells, and decreased astrocyte morphological complexity following stroke in mice [53]. Protein disulfide isomerases (PDIs) are redox chaperones that catalyze the formation or isomerization of disulfide bonds in proteins. In TBI model mice, TBI-induced increased GFAP protein expression was attenuated in PDI3^−/−^ mice [54]. These results suggest that chaperone proteins control TBI-induced conversion of reactive astrocytes through regulation of GFAP expression. 

## 5. Roles of Astrocytes in the Pathogenesis of TBI

Astrocytes can elicit both protective and deleterious actions that influence the repair or aggravation of TBI. TBI-induced secondary injury includes disruption of the BBB, brain edema, and neuroinflammation. On the other hand, neurogenesis, synaptogenesis, and angiogenesis are also promoted to support functions lost in TBI. In this section, the roles of astrocytes in several secondary injuries and neuronal repair are summarized in Table 2.

### 5.1. Neurogenesis and Synaptogenesis

Neurogenesis is promoted in TBI models to replace neurons lost by injury [74]. Astrocytes provide structural and functional support for the proliferation, differentiation, and maturation of neural stem cells [75]. Some potential mechanisms of astrocyte-induced neurogenesis have been proposed in the TBI model. Astrocytes produce the neurotrophic and mitogenic protein S100β. S100β enhances neurogenesis within the hippocampus and improves cognitive function recovery following TBI, and these improvements are mediated by the facilitation of neuronal differentiation, proliferation, and survival of hippocampal progenitor cells [55]. Additionally, adenylate cyclase-activating peptide expressed in astrocytes supports and maintains new neurons after TBI [56]. These observations suggest a role in promoting neurogenesis in astrocytes. However, a contradictory study has been reported. Mice lacking GFAP and vimentin showed increased hippocampal neurogenesis and axonal regeneration post-TBI, suggesting their role in suppressing neurogenesis by astrocytes [16].

Astrocytes also play a crucial role in synaptogenesis after TBI [10]. In the chronic phase after TBI, promoting astrocyte proliferation and increasing the release of astrocyte-derived neurotrophic factors promoted synaptic remodeling in CCI-induced TBI model rats [57]. Astrocytic ephrin-B1, a regulating factor of synapse development in neurons, also induces synapse remodeling through the activation of STAT3-mediated signaling [58]. In contrast, astrocyte-specific elimination of the d-serine-synthesizing enzyme improved synaptic plasticity, brain oscillations, and learning behavior after CCI in mice [59]. The roles of astrocytes that promote or attenuate neurogenesis and synaptogenesis may depend on the brain area or stage of injury, and more detailed roles of astrocytes in neurogenesis and synaptogenesis in TBI need to be elucidated.

### 5.2. BBB Disruption and Angiogenesis

Astrocytes control BBB function by astrocytic end-feet around endothelial cells and astrocyte-derived bioactive factors. BBB dysfunction is commonly observed in both TBI patients and animal models [30,76,77,78,79]. BBB disruption causes vasogenic edema, which results in ICP elevation. Reactive astrocytes secrete multiple bioactive factors that promote BBB disruption and recovery. In TBI model mice, expression of VEGF-A and matrix metalloproteinase-9 (MMP-9), which promote BBB permeability, was increased in reactive astrocytes, and these inhibitions alleviated BBB disruption after TBI [30,41]. Astrocyte-derived ET-1 aggravated BBB disruption, and ET receptor antagonists such as bosentan and BQ788 alleviated BBB disruption in FPI-induced TBI model mice [30,34,60].

In contrast, some astrocyte-derived factors promote angiogenesis and BBB repair. Astrocyte-derived neurotrophic factors alleviate BBB disruption in mice with TBI [61]. Astrocyte-derived fatty acid-binding protein 7 also protected BBB integrity through a caveolin-1/MMP signaling pathway following TBI [62]. Additionally, we found that expression of angiopoietin-1 (Ang-1), which promotes angiogenesis, was increased in astrocytes after TBI in mice, and recombinant Ang-1 administration alleviated TBI-induced BBB disruption [78]. Sonic hedgehog (Shh) is an essential factor in several processes during the development of the vertebrate central nervous system and promotes angiogenesis. Our recent study suggested that expression of Shh was increased in astrocytes after TBI, and administration of exogenous Shh alleviated TBI-induced BBB disruption, whereas Jervine, a Shh inhibitor, aggravated BBB disruption in TBI mice [60]. Salman et al. showed that the Shh pathway was also upregulated in primary human astrocytes following hypoxia while hypothermia inhibited the hypoxia-induced pathway [80]. This may explain why hypothermia has failed in treating stroke since it may inhibit this essential pathway. 

Apolipoprotein-E (APOE) is a protein produced primarily by astrocytes and serves as a major lipid transport molecule in the central nervous system [81]. The E4 variant of APOE (APOE4) is known as a main susceptibility gene for Alzheimer’s disease and leads to accelerated breakdown of the BBB [82]. APOE4 mice displayed prolonged BBB dysfunction compared to APOE3 mice following TBI [81]. Thus, APOE4 is a risk factor for TBI-induced BBB disruption. 

Recent studies suggest several novel methods for evaluating BBB function. Wevers et al. demonstrated successful integration of a human BBB microfluidic model in a high-throughput plate-based format [83]. Additionally, Salman et al. described the design and implementation of an in vitro microvascular open model system using human brain microvascular endothelial cells [84]. The use of humanized self-organized models, organoids, 3D cultures, and human microvessel-on-a-chip platforms must help the development of research on BBB function after TBI. 

### 5.3. Cytotoxic Edema

Cytotoxic edema is characterized by cell swelling due to excessive water retention in brain cells, such as astrocytes, and is observed in the injured brain after TBI. Excessive water accumulation as cytotoxic edema causes an increase in brain water content and elevation of the ICP, leading to irreversible brain injury or death by hernia. Aquaporin-4 (AQP-4) controls the brain water content and is predominantly expressed in astrocytes. AQP-4 is responsible for the formation of cytotoxic edema resulting from excessive astrocyte swelling in TBI. In vitro, FPI increased AQP-4 expression and induced swelling in cultured astrocytes [63]. AQP-4 siRNA alleviated cytotoxic edema in TBI rats, demonstrating the beneficial effects of reduced AQP-4 expression during cytotoxic edema induced by TBI [64]. TBI stimulated nuclear translocation of Foxo3a in astrocytes and upregulated expression of AQP-4, and depletion of Foxo3a rescued cytotoxic edema by preventing the induction of AQP-4 after TBI in mice [65]. Kitchen et al. showed that swelling of the brain or spinal cord is associated with not only total AQP-4 expression but also AQP-4 subcellular translocation to the blood–spinal cord barrier (BSCB) [85]. Calmodulin directly binds the AQP-4 carboxyl terminus, driving AQP-4 cell-surface localization, and inhibition of calmodulin in a rat spinal cord injury model with trifluoperazine, a phenothiazine antipsychotic medicine, inhibited AQP-4 localization to the BSCB, reduced edema, and led to accelerated functional recovery compared with untreated animals [85]. As AQP-4 cell surface expression is controlled by calcium/protein kinase A/calmodulin in astrocytes [85,86], targeting these pathways may also be new therapeutic approaches to treating cytotoxic edema.

Beyond AQP-4, some functional molecules in astrocytes are also considered as initiators of cytotoxic edema formation. Na(+)-K(+)-2Cl(-)-cotransporter 1 (NKCC1) controls the ion gradient by transporting sodium, potassium, and chloride into cells, and is also involved in TBI-induced astrocyte swelling. Cultured astrocytes exposed to trauma by FPI caused a significant increase in NKCC1 activity, and silencing NKCC1 with siRNA led to a reduction in trauma-induced cell swelling [49,66]. Sulfonylurea receptor 1–transient receptor potential melastatin 4 (Sur1-Trpm4) is a cation channel that is upregulated in astrocytes following TBI [19,67]. Blockade of Sur1-Trpm4 reduced CCI-induced edema formation in rats [67]. Therefore, these functional molecules in astrocytes target therapeutic drugs for cytotoxic edema in TBI. 

### 5.4. Neuroinflammation

Neuroinflammation is an innate physiological protective response to infection and injury. However, excessive and chronic inflammation drives neuronal and vascular dysfunction. In both TBI patients and animal models, neuroinflammation is commonly observed [77,87,88,89]. Reactive astrocytes contribute to the inflammatory response of TBI by secreting cytokines, chemokines, nitric oxide, danger-associated molecular patterns, and MMP-9 [68,69,70]. Additionally, astrocytes promote the activation of microglia and immune cells, which induce persistent neuroinflammation. Astrocytes are one of the main producers of IL-33, and IL-33 promotes the recruitment of microglia/macrophages in TBI mice [71]. Astrocyte-derived S100β is also related to neuroinflammation, and S100β knockout mice or administration of the neutralizing S100β antibody significantly reduced TBI-induced microglial activation [72]. Additionally, recent studies have suggested that some microRNAs (miRNAs) regulate neuroinflammation. In the human perilesional cortex, miR155 is most prominently expressed in activated astrocytes, and miR155 promotes inflammation via astrocyte activation after TBI [70]. 

Contrary observations regarding the roles of astrocytes in neuroinflammation have also been suggested. Ablation of reactive astrocytes after moderate CCI in transgenic mice causes more severe inflammation [31]. Additionally, astrocyte-derived exosomes enriched with miR-873a-5p inhibited excessive neuroinflammation by promoting conversion to protective M2 microglia by inhibiting the NF-κB signaling pathway in TBI mice [73]. These results indicate the role of astrocytes in suppressing neuroinflammation. Thus, astrocytes are key players and play dual roles in neuroinflammation.

## 6. Candidate Drugs for Controlling Reactive Astrocytes in TBI

Many candidate drugs have been examined and exert protective actions in TBI model animals. Some of these have also been examined in clinical trials. The candidate drugs are summarized in Table 3. In TBI models, statins, including atorvastatin, lovastatin, and simvastatin, which are inhibitors of 3-hydroxy-3-methylglutaryl coenzyme A reductase and therapeutic drugs for hyperlipidemia, reduced proinflammatory cytokine production and cerebral edema formation [90,91]. A clinical trial in TBI patients demonstrated an improved functional outcome without reducing contusion [92]. Erythropoietin (EPO), a secreted glycoprotein, has also been investigated as a potential therapeutic intervention for TBI, and EPO demonstrated neuroprotective actions in preclinical animal models of TBI [93,94]. However, double-blind randomized patients with TBI showed no evidence of EPO efficiency for neurological outcome at 6 months [95,96]. Mesenchymal stromal/stem cell (MSC) implantation may be a promising strategy for the treatment of TBI. Implantation of SB623, an allogeneic modified bone marrow-derived MSC, appeared to be safe, and TBI patients implanted with SB623 experienced a significant improvement in motor status at 6 months compared to controls [97]. 

Several candidate drugs may target astrocytes. Bumetanide inhibits NKCC1 and reduces astrocytic swelling in vitro after FPI [49]. In an in vivo TBI model, bumetanide reduced astrocytic swelling and BBB disruption [98,99]. Glibenclamide blocks the Sur1-Trpm4 channel that is expressed in astrocytes and reduces regional edema, ICP, hemorrhage, and BBB disruption and improves neurologic dysfunction in TBI models [67,100,101]. In a clinical TBI trial, glibenclamide improved outcomes after moderate-to-severe diffuse axonal injury, but its effect on edema was not evaluated [102]. Another clinical trial demonstrated that glibenclamide reduced contusion expansion but did not influence clinical outcomes in moderate-to-severe TBI [103]. Estrogens such as 17β-estradiol (E2) and progesterone are known as neuroprotective hormones, and estrogen receptors are also highly expressed in astrocytes [119,120]. E2 treatment significantly inhibited excessive astrocyte activation and alleviated neurological deficits, neuronal injuries, and brain edema in rodent TBI models [104,105]. Progesterone administration also decreased lesions, neuronal loss, edema, and improved cognitive function in TBI animals [106]. Protective actions of estrogens include attenuation of neuronal apoptosis, glutamate excitotoxicity, oxidative stress, enhanced release of neurotrophic factors, and suppressing the release of inflammatory cytokines [121,122,123]. However, estrogen administration did not show beneficial effects for TBI in Phase I to III clinical trials [107,108,109]. 

**Figure 2:**
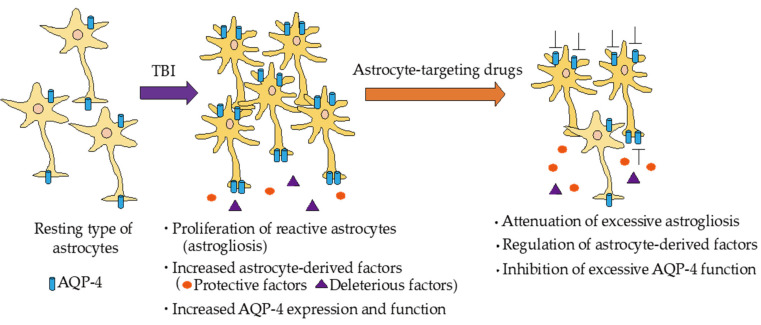
Responses of astrocytes in TBI and expected actions of the astrocyte-targeting drugs. Resting type of astrocyte converts to reactive type in TBI, resulting in induction of astrogliosis. Reactive astrocytes secrete multiple bioactive factors that exert protective and deleterious actions in central nervous tissue in TBI. In addition, expression of aquaporin-4 (AQP-4) is increased in reactive astrocytes, resulting in the promotion of cytotoxic edema formation. Astrocyte-targeting drugs may attenuate excessive astrogliosis, increase protective factors, decrease deleterious factors, and inhibit excessive AQP-4 function.

As astrocytes play multiple roles in TBI pathogenesis, astrocyte-targeting drugs are expected to be novel therapeutic drugs for TBI (Figure 2). Recent studies suggest that several novel candidates exert beneficial effects in experimental TBI models. AER-271, a selective AQP-4 antagonist, showed decreased ICP in a combined model of CCI and hemorrhagic shock [110]. On the other hand, it cannot be denied that AER-271 may have AQP-4-independent effects on brain water transport. Thus, many studies should be performed to validate the AQP-4-dependent effects of AER-271 in future. As AQP-4 is predominantly expressed in astrocytes, selective AQP-4 antagonists may be astrocyte-targeting drugs. Additionally, trifluoperazine, a licensed phenothiazine antipsychotic medicine, inhibited AQP-4 localization to the BSCB, reduced edema, and led to accelerated functional recovery, suggesting a novel candidate drug for cytotoxic edema [85]. Sylvain et al. also suggested that trifluoperazine effectively reduced cerebral edema during the early acute phase in post-stroke mice using a photothrombotic stroke model [111]. Peroxisome proliferator-activated receptors (PPARs) play a critical physiological role in immune responses, and activation of PPARs exerts anti-inflammatory effects, including attenuation of pro-inflammatory mediators. The PPARα receptor agonist fenofibrate reduces post-traumatic neuroinflammation, oxidative stress, cerebral edema, and improved neurological function in TBI models [112,113]. Similarly, the PPARγ receptor agonists pioglitazone and rosiglitazone also improved functional and histological outcomes after TBI [114,115,116]. As astrocytes highly express PPARγ [124,125], the beneficial actions of PPARγ agonists may be through controlling reactive astrocytes. SB-3CT is a highly selective inhibitor of MMP-2 and MMP-9, and SB-3CT showed promising results in preclinical models of TBI by FPI and CCI. SB-3CT reduced lesion volume, microglial activation, and astrogliosis after TBI [117,118]. However, the time and degree of MMP inhibition must be cautious because MMPs also contribute to neurovascular remodeling and repair [126,127]. Additionally, we suggest that endothelin ET_B_ receptors are predominantly expressed in reactive astrocytes after TBI in mouse cerebrum, and administration of BQ788, a selective ET_B_ receptor antagonist, reduced the increase in reactive astrocytes [30]. Additionally, BQ788 alleviated BBB disruption and brain edema by decreasing astrocytic MMP-9 and VEGF-A expression, and increased astrocytic ANG-1 and SHH expression in TBI mice [30,60,78]. Bosentan, a non-selective ET receptor antagonist that is used to treat pulmonary arterial hypertension in the clinical state, also ameliorated TBI-induced BBB disruption and brain edema in mice [34]. Although these drugs have not been examined in clinical trials for TBI patients, they may be novel candidates for therapeutic drugs for TBI by controlling the functions of reactive astrocytes. 

## 7. Conclusions

Emerging studies suggest that the responses and roles of astrocytes in TBI are extremely complicated and controlled by multiple bioactive factors and intracellular signaling mechanisms. Although reactive astrocytes exert different actions in TBI, these actions may depend on the severity, stage, and brain area. As shown in Table 2, reactive astrocytes have multiple functions in TBI, including promotion and restriction of neurogenesis and synaptogenesis, acceleration and suppression of neuroinflammation, disruption and repair of the BBB, and regulation of brain edema formation. These facts imply that astrocytes are widely involved in TBI pathogenesis and are key players for therapy of TBI. Thus, selective stimulation of astrocytic beneficial functions and attenuation of astrocytic deleterious functions are promising astrocyte-targeting therapeutic strategies. Increased astrocyte-derived neurotrophic factors and vascular protective factors promote recovery of neuronal function and BBB while decreased astrocyte-derived inflammatory factors suppress neuroinflammation in TBI. 

Astrocytic AQP-4 is an attractive target for TBI-induced cytotoxic edema. Upregulation of AQP-4 occurs at the site of TBI while downregulation of AQP-4 occurs adjacent to the site of injury [128]. Inhibition of astrocytic AQP-4 reduces the TBI-induced cytotoxic edema described in Section 5.3. During edema formation, astrocytic AQP-4 has been shown to facilitate cytotoxic edema by astrocyte swelling while AQP-4 has also been seen to be responsible for the reabsorption of extracellular edema fluid, resulting in reduction of vasogenic edema [129]. Therefore, the timing of AQP-4 inhibition is important for therapy for brain edema in TBI. Previous studies showed an increased AQP-4 membrane localization in astrocytes which was not accompanied by a change in AQP-4 protein expression levels [130]. As Ciappelloni et al. suggest that trafficking AQP-4 to membrane surface controls astrocytic function [131], understanding in detail the mechanisms of trafficking astrocytic AQP-4 to the cell surface may help in the development of new treatments for TBI-induced cytotoxic edema.

A large number of animal experiments have been performed to develop novel therapeutic drugs for TBI. However, most of them have failed to show beneficial effects in patients with TBI in clinical trials. These candidate drugs mainly target neuronal cells or cerebrovascular diseases. Because astrocytes also play a key role in the pathogenesis of TBI, astrocyte-derived bioactive factors and astrocytic functional molecules are attractive targets. Several candidate drugs described in this review may target astrocytes and control the function of reactive astrocytes. Recently, the use of high-throughput screening and computer-aided drug design were reviewed by Aldewachi et al. [132] and Salman et al. [133]. These methods must support the discovery of novel drugs. Although we should elucidate the specific roles of astrocytes and the mechanisms regulating TBI pathophysiology by astrocytes, additional potential therapeutic targets for astrocytes must emerge in the future. 

## Figures and Tables

**Table 1:** Summary of the endogenous bioactive regulators for astrogliosis.

Factors	Related Receptors	Effects	References
ET-1	ET_B_ receptor	ET_B_ receptor antagonist reduced conversion to reactive astrocytes in TBI mice.	[30]
IL-1	IL-1 receptor	IL-1 promoted conversion to reactive astrocytes. IL-1 receptor antagonist reduced astrogliosis in TBI mice.	[36,37]
MCP-1	CCR5	The CCR5 knockdown reduced astrogliosis in TBI in mice. Pharmacological CCR5 antagonist reduced astrogliosis in TBI mice.	[38,39,40]
VEGF	VEGF receptor	VEGF inhibitor reduced reactive astrocytes after TBI in mice.	[41]

**Table 2:** Summary for roles of the reactive astrocytes in TBI.

TBI Pathogenesis	Promoting Effects	Suppressing Effects
Neurogenesis Synaptogenesis	Astrocyte-derived factors promoted neurogenesis in TBI animals [55,56]. Astrocyte-derived neurotrophic factors promoted synaptic remodeling in TBI animals [57,58].	Mice lacking GFAP and vimentin showed increased hippocampal neurogenesis and axonal regeneration in TBI animals [16]. Astrocyte-specific elimination of d-serine-synthesizing enzyme improved synaptic plasticity in TBI animals [59].
BBB disruption	Astrocyte-derived ET-1, VEGF, and MMP-9 promoted BBB disruption [30,34,41,60].	Astrocyte-derived neurotrophic factors, fatty acid-binding protein 7, Ang-1, and Shh suppressed BBB disruption in TBI mice [60,61,62].
Cytotoxic edema	FPI-induced increase in AQP-4 expression promoted swelling in cultured astrocytes [63]. AQP-4 siRNA alleviated cytotoxic edema in TBI animals [64]. Depletion of Foxo3a rescued cytotoxic edema by preventing induction of AQP-4 in TBI animals [65]. NKCC1 siRNA reduced trauma-induced cell swelling in cultured astrocytes [49,66]. Blockade of Sur1-Trpm4 reduced edema formation in TBI animals [67].	
Neuroinflammation	Reactive astrocytes contributed to neuroinflammation by secreting cytokines, chemokines, nitric oxide, danger-associated molecular patterns, and MMP-9 [68,69,70]. Astrocyte-derived IL-33 promoted recruitment of microglia/macrophages in TBI animals [71]. S100β knockout mice or administration of neutralizing S100β antibody significantly reduced microglial activation in TBI animals [72]. MiR155 promoted brain inflammation via astrocyte activation after TBI [70].	Ablation of reactive astrocytes caused more severe inflammation in TBI animals [31]. Astrocyte-derived exosomes enriched with miR-873a-5p inhibited neuroinflammation [73].

**Table 3:** Summary of the candidate drugs for TBI.

Candidate Drugs	Preclinical Effects	Clinical Trials	References
Statins (atorvastatin, lovastatin, simvastatin)	Statins reduced proinflammatory cytokine production and cerebral edema formation in TBI animals.	Clinical trial demonstrated an improved functional outcome, but without reducing contusion.	[90,91,92]
Erythropoietin	Erythropoietin demonstrated neuroprotective efficacy in TBI animals.	Clinical trials showed no evidence of EPO efficiency for neurological outcome.	[93,94,95,96]
SB623 (allogeneic modified bone marrow-derived MSCs)		Implantation of SB623 showed significant improvement of motor status.	[97]
Bumetanide (NKCC1 inhibitor)	Bumetanide reduced astrocytic swelling in vitro after FPI. Bumetanide reduced cellular swelling and BBB disruption in TBI animals.	Not performed.	[66,98,99]
Glibenclamide (Sur1-Trpm4 channel inhibitor)	Glibenclamide reduced edema, ICP, hemorrhage, BBB disruption, and improved neurologic dysfunction in TBI models.	Glibenclamide improved outcomes after moderate-to-severe diffuse axonal injury while the effect on edema was not evaluated. Glibenclamide reduced contusion expansion but did not influence clinical outcome in moderate-to-severe TBI.	[67,100,101,102,103]
Estrogens (17β-estradiol, progesterone)	17β-estradiol inhibited excessive astrocyte activation and alleviated neurological deficits, neuronal injuries, and edema in rodent TBI models. Progesterone decreased lesions, neuronal loss, and edema and improved cognitive function in TBI animals.	Estrogens did not show beneficial effects for TBI in Phase I to III clinical trials.	[104,105,106,107,108,109]
AER-271 (selective AQP-4 antagonist)	AER-271 showed a decreased ICP in a combined model of CCI and hemorrhagic shock.	Not performed.	[110]
Trifluoperazine (phenothiazine antipsychotic medicine)	Trifluoperazine inhibited AQP-4 localization to the BSCB, reduced edema, and led to accelerated functional recovery.		[86,111]
Fenofibrate (PPARα agonist) Pioglitazone, rosiglitazone (PPARγ agonist)	Fenofibrate reduced neuroinflammation, oxidative stress, and cerebral edema, and improved neurological function in TBI models. Pioglitazone and rosiglitazone improved functional and histological outcomes in TBI animals.	Not performed.	[112,113,114,115,116]
SB-3CT (selective MMP-2 and -9 inhibitor)	SB-3CT reduced lesion volume, microglial activation, and astrogliosis after TBI animals.	Not performed.	[117,118]
BQ788 (selective ET_B_ receptor antagonist) Bosentan (non-selective ET receptor antagonist)	BQ788 decreased in excessive reactive astrocytes, alleviated the BBB disruption and brain edema in TBI animals. Bosentan ameliorated BBB disruption and brain edema in TBI animals.	Not performed.	[30,34,60,78]
